# Supplementary material for: Enhancing the Solubility and Dissolution of Apigenin: Solid Dispersions Approach
Source: Int J Mol Sci. 2025 Jan 10;26(2):566. doi: 10.3390/ijms26020566 (PMC11766082; doi:10.3390/ijms26020566)
Supplement: Supplementary file 1 [file ijms-26-00566-s001.zip › ijms-3362640-supplementary.pdf]

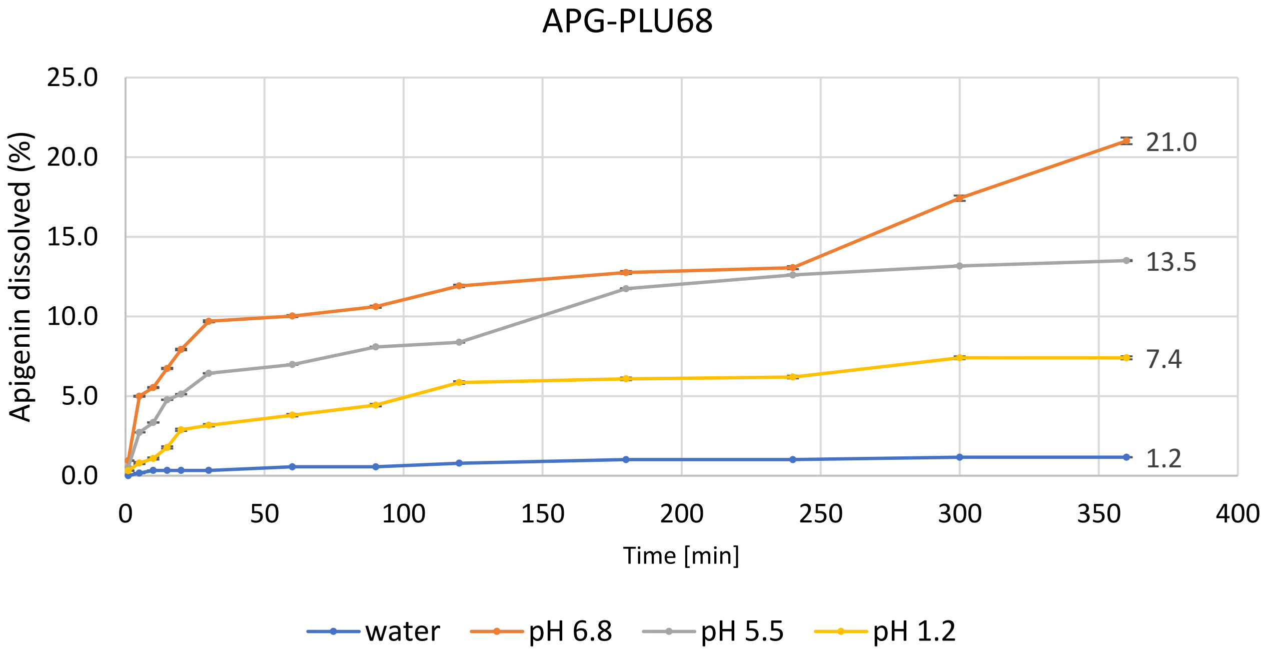

(a)

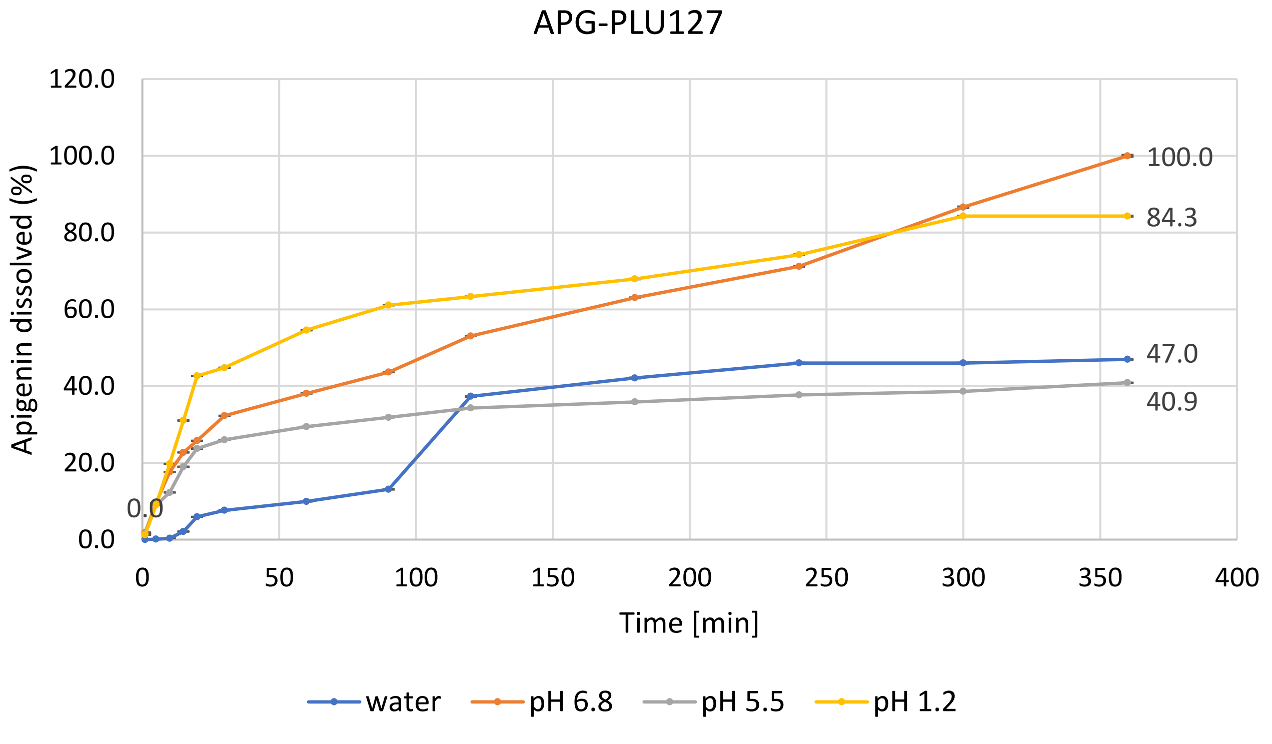

(b)

Figure S1. Apigenin release profiles from (a) APG-PLU68 and (b) APG-PLU127 solid dispersions: in water (blue), pH 6.8 buffer (orange), pH 5.5 buffer (green) and pH 1.2 buffer (yellow).

Table S1. Assignment of vibrational modes in the APG molecule to individual FT-IR spectrum peaks [20].

**Band theoretical [cm^{-1}]**	**Band experimental [cm^{-1}]**	**Band assignment**
**3119**	3281	ν_{CH}
**3088**	3096	ν_{CH}
**1652**	1651	$\nu_{OC} + \nu_{CC}$
**1610**	1605	$\nu_{OC} + \nu_{CC}$
**1587**	1587	$\nu_{CC} + \nu_{OC}$
**1563**	1557	$\nu_{CC} + \delta_{HOC}$
**1500**	1495	$\delta_{HCC} + \nu_{CC}$
**1486**	1472	$\delta_{HCC} + \delta_{HOC}$
**1450**	1456	$\delta_{HCC} + \nu_{OC} + \delta_{CCC}$
**1424**	1443	$\delta_{HCC} + \nu_{CC} + \delta_{HOC}$
**1418**	1400	$\delta_{HOC} + \delta_{HCC}$
**1342**	1352	$\nu_{OC} + \delta_{HOC}$
**1281**	1296	$\delta_{CCC} + \nu_{CC}$
**1272**	1269	$\nu_{OC} + \delta_{HCC}$
**1249**	1244	$\delta_{HCC} + \nu_{CC}$
**1216**	1221	$\nu_{CC} + \delta_{HOC} + \delta_{HCC}$
**1161**	1177	$\delta_{HOC} + \delta_{HCC}$
**1152**	1155	$\delta_{HCC} + \nu_{OC}$
**1136**	1117	$\delta_{HCC} + \delta_{HOC}$
**1098**	1105	$\delta_{HCC} + \delta_{HOC}$
**1009**	1032	δHCC + νCC
**908**	908	τHCCC + τCCCC
**825**	827	τCCCC
**808**	806	τHCCC
**741**	737	τHCCC + γOCCC
**704**	692	δCCC + γOCCC
**666**	669	γOCCC
**645**	648	δCCC + δCOC
**635**	633	γHOC + τHCCC
**591**	579	γOCCC
**561**	567	δCCC
**499**	501	γCCOC + γOCCC
**475**	473	δCOC + δCCC + δOCC
**425**	453	δCCC

Legend: ν – stretching vibrations; δ – in-plane bending vibrations; γ – out-of-plane bending vibrations; τ – torsional vibrations
